# Application of a machine learning model based on routine clinical parameters for the diagnosis of rheumatoid arthritis with concomitant osteoporosis: a retrospective study

**DOI:** 10.7717/peerj.20852

**Published:** 2026-02-27

**Authors:** Zhe Wu, Zhaohui Li, Weifeng Li, Shengren Xiong

**Affiliations:** Orthopedics, Fujian Medical University Union Hospital, Fuzhou, China

**Keywords:** Rheumatoid arthritis, Osteoporosis, Routine clinical parameters, Machine learning model

## Abstract

Rheumatoid arthritis (RA) is commonly complicated by secondary osteoporosis (OP), affecting up to 80% of patients. Although dual-energy X-ray absorptiometry (DEXA) is the diagnostic gold standard, its limited accessibility highlights the need for alternative tools. In this retrospective cohort study of 396 hospitalized RA patients, we developed machine learning models using demographic and routine laboratory data to identify concomitant OP. Five classifiers were evaluated and combined *via* a soft-voting ensemble. The support vector machine achieved the highest area under the curve (AUC) (86.5%), while the random forest showed the highest accuracy (81.5%). The ensemble model demonstrated balanced performance (AUC 83.2%; accuracy 81.1%). SHapley Additive exPlanations (SHAP) analysis indicated age, sex, and body mass index (BMI) as major contributors, whereas albumin and inflammatory markers—including platelet-to-lymphocyte ratio (PLR), neutrophil percentage/albumin ratio (NPAR), white blood cell count (WBC), and neutrophil-to-lymphocyte ratio (NLR)—showed modest but heterogeneous influences on model predictions. These findings suggest that machine learning models incorporating routinely collected clinical data offer a practical and interpretable approach for preliminary OP risk assessment in RA. However, given the single-center design and limited sample size, the results should be considered exploratory, and larger external validation studies are warranted.

## Introduction

Rheumatoid arthritis (RA) is a typical chronic autoimmune disease characterized by erosive synovitis, with a global prevalence of approximately 0.5–1% (Leng et al., 2024). RA not only affects the joints but also leads to multi-system pathological changes, particularly in bone metabolism. Epidemiological data show that approximately 30–50% of RA patients will eventually develop secondary osteoporosis (OP), with the incidence of OP being 1.8 times higher than that in the general population (Adami & Saag, 2019). In clinical practice, early diagnosis and osteoporosis risk assessment are crucial for the treatment and prognosis of patients (Chen, Huang & Chen, 2022). Dual-energy X-ray absorptiometry (DEXA), widely regarded as the gold standard for OP diagnosis (Wu et al., 2023), remains limited in its clinical utility due to high equipment cost, restricted availability, and radiation-related concerns, which hinder its widespread use, especially in primary care settings (Baik et al., 2024; Kwon et al., 2022; Yang et al., 2023).

From a pathophysiological perspective, the development of RA-related osteoporosis is influenced by multiple interacting factors (Kareem et al., 2021; Song et al., 2022; Westhovens & Dequeker, 2000; Yu et al., 2021). Both RA and OP exhibit significant gender differences, with a notably higher prevalence in females (Inui et al., 2023). Inflammation and nutritional disturbances play essential roles in bone loss (Al Salmani et al., 2022; Demir Cendek et al., 2024; Liu et al., 2022), and serum albumin (ALB)—a marker reflecting both inflammation and nutritional status—has been associated with chronic disease severity (Cao et al., 2021). Emerging inflammatory markers such as the neutrophil-to-lymphocyte ratio (NLR) (Liu et al., 2023; Salimi et al., 2024), platelet-to-lymphocyte ratio (PLR) (Gao et al., 2019), and neutrophil percentage/albumin ratio (NPAR) have gained wide clinical application due to their ease of measurement (Duan et al., 2024; Tang et al., 2022; Zhou et al., 2023). Demographic and clinical indicators such as age, sex, and body mass index (BMI) also influence bone metabolism and are routinely measured in hospitalized patients, offering multidimensional information on individual health status (Lee et al., 2023).

Artificial intelligence has been rapidly incorporated into rheumatological assessment in recent years. Beyond laboratory-based risk modeling, significant advances have occurred in artificial intelligence (AI)-assisted imaging diagnostics. In the domain of musculoskeletal ultrasound (US), Fiorentino et al. (2019) developed a convolutional-neural-network–based framework for informative-frame selection in rheumatology US examinations, enabling automatic identification of diagnostically relevant images and reducing operator dependence. Building on this, their 2022 work proposed a deep-learning model for automated estimation of metacarpal-head cartilage thickness, providing a reproducible, non-invasive tool to quantify early structural joint changes (Fiorentino et al., 2022). These imaging-oriented models represent an important direction in AI applications, but they rely on specialized equipment, standardized US acquisition, and expert annotation, which may limit their accessibility—particularly in community hospitals and primary care settings.

Despite the recognized relevance of these routinely collected clinical variables, existing RA-related osteoporosis risk assessment still relies heavily on DEXA or simple demographic stratification and lacks practical tools that integrate inflammatory and nutritional biomarkers into a unified risk-prediction framework. Although machine learning (ML) has been increasingly applied in the medical field (Lin et al., 2022; Zhao et al., 2024), enabling the extraction of complex patterns from large datasets (Forrest et al., 2023; Solomon et al., 2023), few ML studies have specifically targeted RA-related osteoporosis, and prior work (*e.g*., Lee et al., 2023) has primarily incorporated demographic or socioeconomic factors rather than RA-specific biological indicators. This gap limits early identification of high-risk patients and restricts the clinical applicability of existing models.

To address this unmet need, the present study utilizes routinely available blood parameters, serum albumin levels, inflammatory indexes, and demographic factors to explore their relationship with secondary osteoporosis in RA using machine learning algorithms (Aitella et al., 2025). These indicators are not only widely accessible, easy to obtain, and cost-effective, but also reflect the patient’s inflammatory and nutritional status, providing a more comprehensive assessment than demographic data alone. The goal is to develop an interpretable, practical, and low-cost predictive model that complements the limitations of DEXA, supports early risk stratification, and facilitates personalized clinical decision-making for RA patients.

The hypothesis and value of this study lie in integrating routine clinical indicators to develop a machine learning predictive model with the following objectives: (1) effectively distinguishing between RA patients and those with RA and OP; (2) providing a simple, low-cost screening tool for RA-related OP in primary care settings; (3) offering evidence-based support for early intervention and personalized treatment for RA patients. Ultimately, this model aims to improve early osteoporosis detection in RA patients and provide data-driven evidence to guide future clinical practice and public health strategies.

## Methods

The study is a retrospective cohort study, with data sourced from the electronic medical records system of the Fujian Medical University Union Hospital between March 1, 2020, and February 28, 2025. We identified patients with rheumatoid arthritis (RA) using ICD-10 codes, reviewed their medical records to confirm that they met the diagnostic criteria for RA, and applied the inclusion and exclusion criteria. A total of 396 cases were included in this study. The study was approved by the Ethics Committee of Fujian Medical University Union Hospital (2025KY119). Informed consent was waived due to retrosopective nature of the study. The study adhered to the Standards for Reporting of Diagnostic Accuracy Studies (STARD) guidelines for diagnostic research reports (Cohen et al., 2016). Patients were divided into two groups: the pure RA group and the RA with osteoporosis (OP) group. Based on the reviewed medical records, patients who had confirmed osteoporotic fractures or whose bone mineral density met the diagnostic criteria for osteoporosis were classified into the RA with OP group.

Inclusion criteria:

1. Age ≥ 18 years.
2. A confirmed diagnosis of rheumatoid arthritis.
3. Patients with osteoporosis must have either a documented osteoporotic fracture or be confirmed by DEXA.

Exclusion criteria:

1. Patients with a history of malignancy prior to the diagnosis of rheumatoid arthritis.
2. Patients with pre-existing osteoporosis before the diagnosis of rheumatoid arthritis.
3. Patients with other concomitant autoimmune diseases.
4. Patients with missing data or incomplete clinical information.

### Statistical analysis

Data analysis for this study was performed using SAS OnDemand for Academics (SAS Institute, Inc., Cary, North Carolina, USA). First, continuous variables (such as age, BMI, and other clinical characteristics) between the rheumatoid arthritis (RA) group and the rheumatoid arthritis with osteoporosis (RA+OP) group were compared using the t-test. Gender, as a categorical variable, was compared between groups using the chi-square test (χ^2^ test). All statistical tests were two-tailed, with a significance level set at *P* < 0.05, indicating that results with a *P*-value less than 0.05 were considered statistically significant. All analyses were conducted using complete datasets to ensure the reliability and accuracy of the results.

### Model development and validation

All machine learning analyses in this study were conducted using Python version 3.12. Five base classifiers were employed: Random Forest, Gradient Boosting, Extreme Gradient Boosting (XG Boost), Support Vector Machine (SVM), and Naive Bayes. The model was not adjusted for treatment exposure. These models were integrated using an ensemble learning approach through a soft voting classifier (Voting Classifier), with model weights assigned as follows: [2, 1, 1, 2, 1]. Weights were assigned based on preliminary cross-validation accuracy, with Random Forest (0.95) and SVM (0.87) receiving higher weights than Gradient Boosting (0.73), XGBoost (0.75), and Naive Bayes (0.68) due to their superior individual performance.

The dataset was randomly split into a training set and a testing set in an 80:20 ratio, ensuring consistent class distribution across both subsets. Prior to model training, data in the training set were standardized using the fit_transform method to prevent information leakage. The same transformation parameters were then applied to the test set using the transform method, ensuring model generalizability to unseen data. Hyperparameter tuning for each base classifier was conducted using GridSearchCV with five-fold cross-validation. The predefined search spaces were as follows: Random Forest (n_estimators: [100, 200, 300], max_depth: [5, 7, 9], min_samples_split: [2, 5, 10]), Gradient Boosting (n_estimators: [100, 200], learning_rate: [0.01, 0.1], max_depth: [3, 5]), XGBoost (n_estimators: [100, 150], learning_rate: [0.01, 0.1], max_depth: [6, 8]), and Support Vector Machine (C: [2, 5], gamma: [‘scale’, ‘auto’]). Gaussian Naïve Bayes was used without hyperparameter optimization due to its parameter-free nature. The primary optimization metric was the area under the receiver operating characteristic curve (ROC AUC), selected to ensure robust performance across multiple subsets of the data. Model training was executed using the fit method, with hyperparameter selection embedded within the training process.

To evaluate model robustness and estimate confidence intervals, bootstrap resampling (*n* = 100) was applied. The 95% confidence intervals for accuracy and AUC were calculated to assess variability and model reliability. Performance metrics reported include precision, recall, and F1-score. Confusion matrices were also visualized to detail the distribution of true positives (TP), true negatives (TN), false positives (FP), and false negatives (FN).

To enhance interpretability and gain insights into the model’s decision-making process, Shapley Additive Explanations (SHAP) were applied. SHAP values quantified the contribution of each feature to the model’s predictions. The results of this interpretability analysis were visualized as bar plots, illustrating the overall impact of individual features on model output.

## Results

### Baseline characteristics

A total of 470 hospitalized patients diagnosed with rheumatoid arthritis (RA) were initially screened. After excluding 74 cases based on predetermined criteria, 396 patients were included in the final analysis. Patients were divided into two groups: RA alone and RA with concomitant osteoporosis (RA+OP).

Comparison of baseline characteristics revealed that both groups were predominantly female; however, the proportion of female patients was significantly higher in the RA+OP group than in the RA-only group (χ^2^ = 28.25; *P* < 0.01). Patients in the RA+OP group were significantly older (mean [SD], 64.60 [8.44] years *vs*. 56.61 [12.37] years; *P* < 0.01) and had lower BMI (21.80 [3.33] *vs*. 22.57 [3.42]; *P* = 0.02) (Table 1).

**Table 1:** The characteristics of the subjects.

		RA (*n* = 197)	RA&OP (*n* = 199)	*P*	Difference (95% CI)
Gender	Men	71 (36.04%)	26 (13.07%)	<0.01	
	Women	126	173		
Age		56.61 ± 12.37	64.60 ± 8.44	<0.01	7.99 [5.82–10.16]
BMI		22.57 ± 3.42	21.80 ± 3.33	=0.02	0.77 [0.10–1.44]
WBC		7.13 ± 2.58	7.13 ± 2.82	=0.99	0.00 [−0.53 to 0.53]
Alb		35.43 ± 4.49	35.65 ± 4.73	=0.64	0.22 [−0.69 to 1.13]
NLR		3.65 ± 2.39	3.68 ± 2.77	=0.90	0.03 [−0.48 to 0.54]
PLR		217.4 ± 115.3	212.6 ± 115.6	=0.68	−4.74 [−27.55 to 18.07]
NPAR		1.94 ± 0.49	1.91 ± 0.48	=0.62	−0.02 [−0.12 to 0.07]

**Note:**

CI, confidence interval

No significant differences were observed between the two groups in other clinical parameters, including white blood cell count (WBC), albumin (Alb), neutrophil-to-lymphocyte ratio (NLR), platelet-to-lymphocyte ratio (PLR), and neutrophil percentage-to-albumin ratio (NPAR) (*P* = 0.99, 0.64, 0.90, 0.68, and 0.62, respectively).

### Model performance

**Figure 1:**
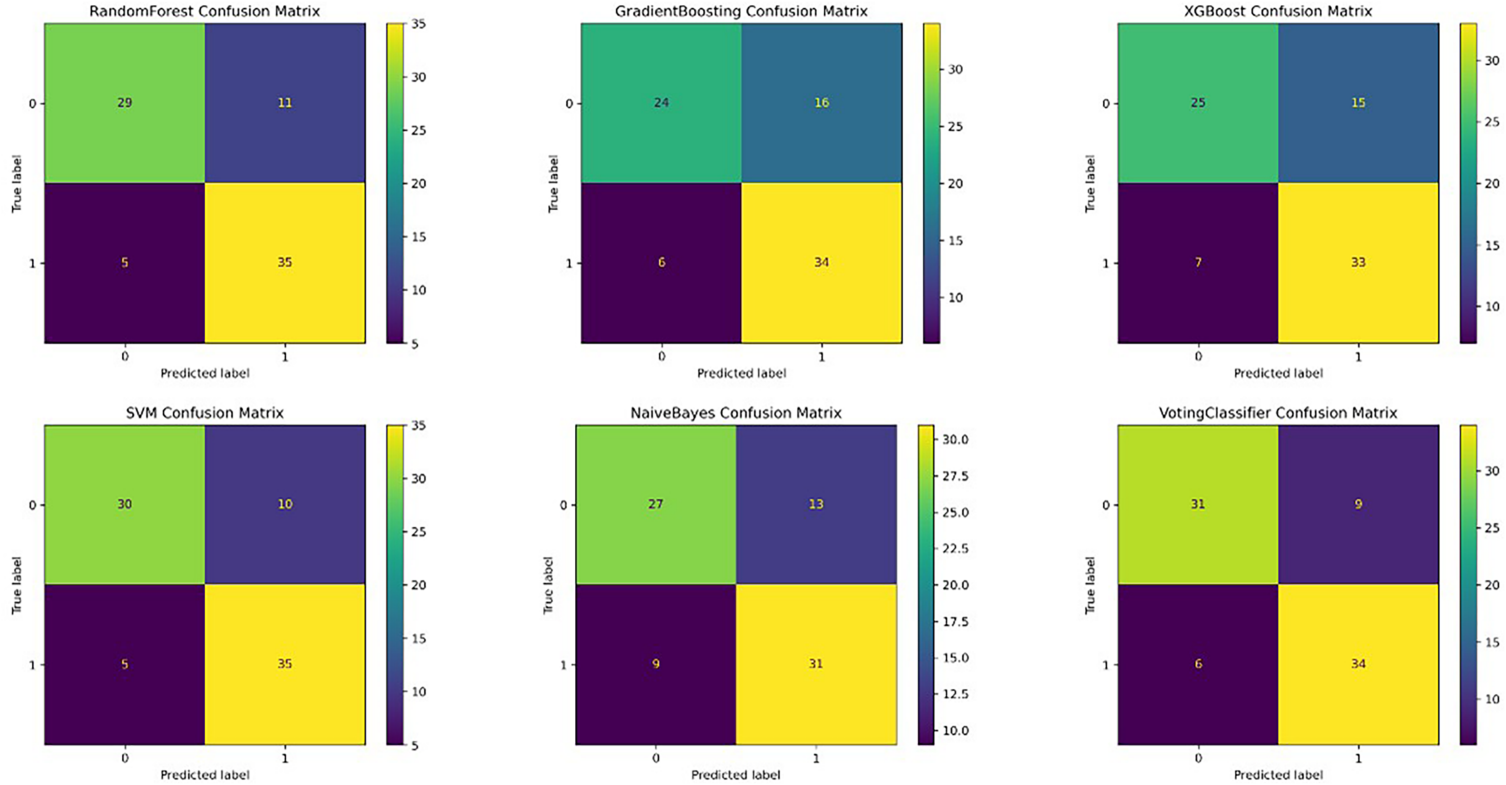
The confusion matrix shows the model performance by indicating the number of rheumatoid arthritis correctly identified with or without osteoporosis in each group.

**Figure 2:**
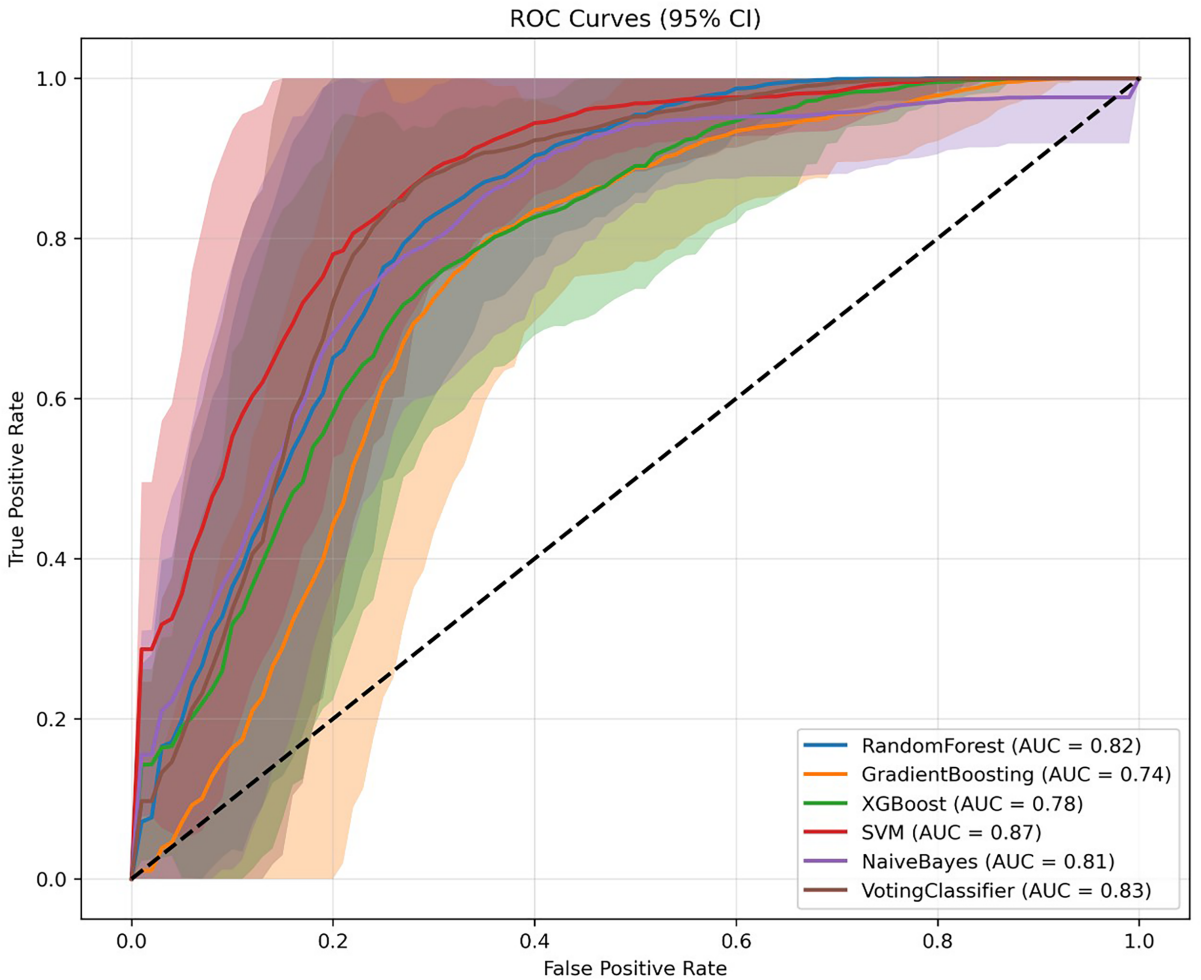
Evaluation of the performance of all machine learning models with ROC in the internal validation cohort.

To evaluate model performance, we compared ensemble learning approaches with traditional classifiers on a balanced testing dataset (40 samples per class). Key performance metrics included accuracy, sensitivity (recall), specificity, and the area under the receiver operating characteristic curve (AUC) (Table 2; Figs. 1, 2).

**Table 2:** Diagnostic performance of models in differentiating two diseases.

Model	Accuracy, % (95% CI)	ROC AUC, % (95% CI)	Sensitivity (Class 1), %	Specificity (Class 0), %
Random forest	81.5 [73.8–88.8]	80.9 [70.4–89.2]	88	86
SVM	81.2 [72.4–90.0]	86.5 [79.7–92.1]	88	75
Voting classifier	81.1 [70.0–88.8]	83.2 [75.9–92.0]	85	78
Gradient boosting	71.9 [63.8–80.0]	73.9 [63.6–84.1]	85	60
XGBoost	72.0 [63.1–80.7]	78.4 [69.4–87.0]	82	62
Naive Bayes	72.5 [61.3–81.3]	80.3 [70.1–88.8]	78	68

### Random forest model

Following hyperparameter tuning (max_depth = 7, n_estimators = 200), the Random Forest model achieved the highest diagnostic accuracy of 81.5% (95% CI, [73.8–88.8%]) and an AUC of 80.9% (95% CI, [70.4–89.2%]). The model demonstrated high specificity for class 0 (precision = 86%) and high sensitivity for class 1 (recall = 88%), suggesting its effectiveness in minimizing false-positive diagnoses for patients without osteoporosis.

### Support vector machine

The SVM model showed comparable accuracy (81.2%; 95% CI, [72.4–90.0%]) but outperformed all other models in terms of AUC (86.5%; 95% CI, [79.7–92.1%]), indicating excellent discriminative ability. It exhibited high sensitivity for class 1 (recall = 88%), similar to the Random Forest model, but lower specificity for class 0 (precision = 75%).

### Voting classifier

The Voting Classifier, which integrated the predictions of multiple base models, achieved balanced performance with an accuracy of 81.1% and AUC of 83.2%. These results suggest that ensemble integration yielded stable predictions by leveraging the strengths of individual models.

### Other models

Gradient Boosting, XGBoost, and Naive Bayes demonstrated lower performance, likely reflecting limited ability to capture nonlinear interactions in the dataset.

### Class-specific trends

Across models, sensitivity for RA+OP was consistently higher than specificity, indicating greater model success in identifying patients requiring osteoporosis screening. This pattern aligns with the clinical priority to avoid missed diagnoses.

In summary, both Random Forest and SVM models demonstrated superior overall performance in terms of accuracy and AUC, with SVM showing a distinct advantage in discrimination capability. The Voting Classifier provided stable and balanced predictions by aggregating model outputs. In contrast, Gradient Boosting and XGBoost showed relatively suboptimal performance and may require further tuning. Naive Bayes, while efficient, exhibited limitations in identifying complex disease characteristics.

### Feature importance *via* SHAP values

**Figure 3:**
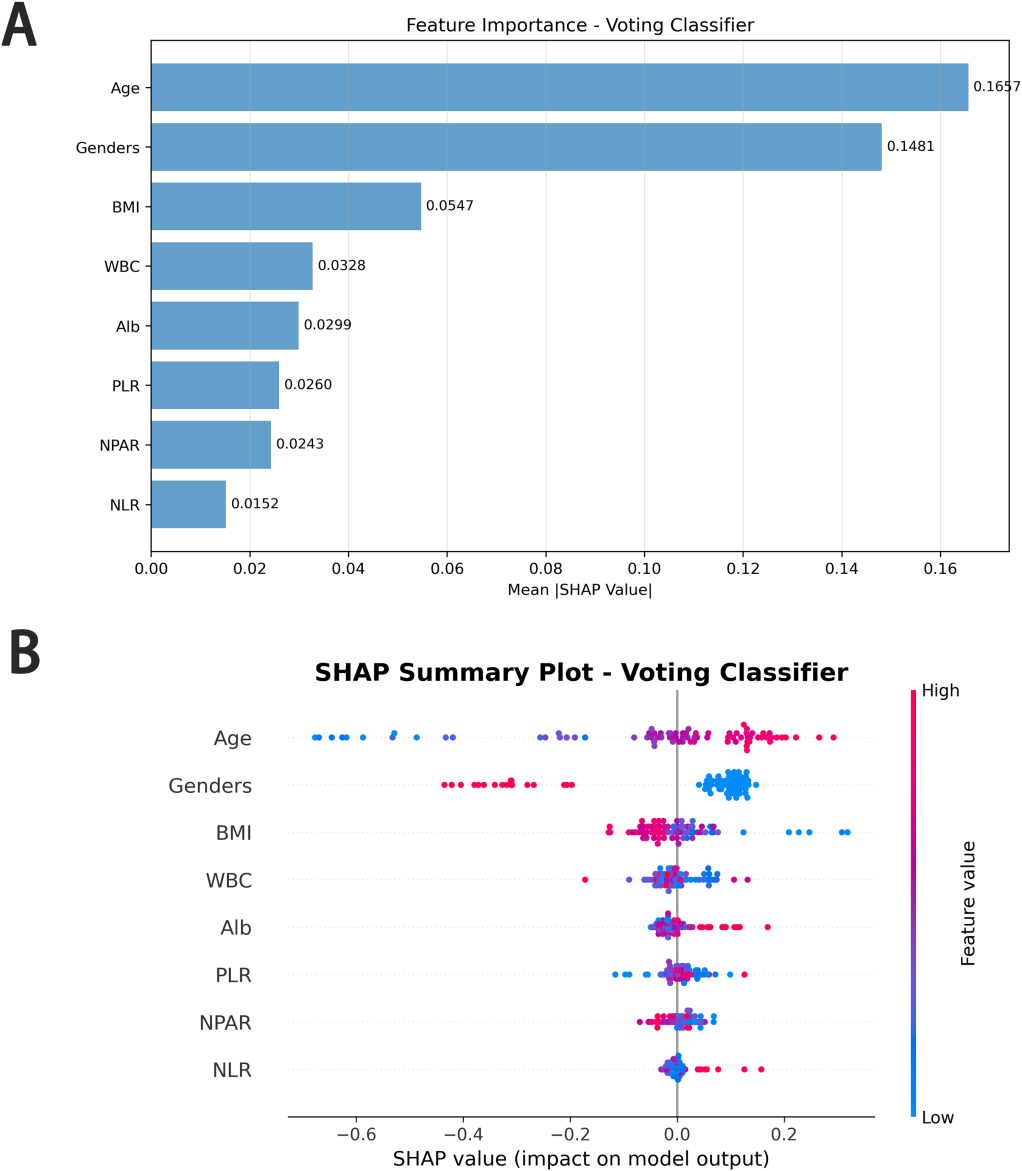
SHAP value and importance for each feature of our support vector machine model. (A) Mean absolute SHAP values ranking the overall contribution of each feature to the model’s predictions for distinguishing rheumatoid arthritis alone (RA) from RA with concomitant osteoporosis (RA+OP). (B) SHAP summary plot displaying the direction and magnitude of each feature’s influence on model output. Each point represents an individual patient, with color indicating feature value (blue = low, red = high).

SHAP analysis was applied to quantify and visualize the contribution of each feature to the prediction of concomitant osteoporosis in patients with RA using the Voting Classifier model. Figure 3 presents both the global feature importance ranking (Fig. 3A) and the distribution of SHAP values across individual patients (Fig. 3B).

As shown in Fig. 3A, age demonstrated the largest mean absolute SHAP value (0.1667), followed by sex (0.1481). BMI ranked third (0.0547), indicating that it was one of the major contributors to model prediction. WBC (0.0328), albumin (Alb; 0.0299), PLR (0.0260), NPAR (0.0243), and NLR (0.0152) showed comparatively smaller contributions.

Figure 3B illustrates the directionality of feature influence. Age and female sex generally yielded positive SHAP values, shifting predictions toward the RA+OP class. BMI exhibited a predominantly negative SHAP distribution at higher values and positive values at lower values, indicating that higher BMI was associated with a decreased model-predicted probability of RA+OP.

For biochemical and inflammatory markers, WBC, Alb, PLR, NPAR, and NLR all showed mixed positive and negative SHAP values with considerable overlap around zero, suggesting no consistent directional influence across the cohort. These features contributed to the model with variable effects at the individual level, rather than demonstrating uniform shifts toward or away from the RA+OP class.

Overall, SHAP analysis clarified both the relative importance and the heterogeneity of the effects of each feature. Age, sex, and BMI were the strongest contributors, while the remaining laboratory features exerted modest and directionally variable influences on the model’s predictions. Laboratory features provide incremental and individualized predictive value, rather than dominant contributions.

## Discussion

In this study, we developed an interpretable ensemble machine learning model based on routinely collected clinical parameters to differentiate patients with rheumatoid arthritis (RA) from those with concomitant osteoporosis (RA+OP). Among the evaluated algorithms, the support vector machine (SVM) achieved the highest AUC, whereas the random forest and voting classifier provided a favorable balance between performance and interpretability. These findings support the potential role of machine learning as a complementary clinical tool for early identification of osteoporosis risk in RA, particularly in settings where DEXA availability is limited. The Fracture Risk Assessment Tool (FRAX) is widely used for fracture risk assessment and the prevention and treatment of osteoporosis. Unlike FRAX, our model incorporates routine laboratory data, making it a complementary tool to FRAX for joint use. It can serve as an initial screening tool for osteoporosis in patients with rheumatoid arthritis.

SHAP analysis provided deeper insight into how individual features contributed to model predictions. Age and sex were the dominant predictors, consistent with the observed demographic differences between groups. BMI ranked third in importance and showed a predominantly protective pattern, with higher BMI associated with lower predicted RA+OP probability. In contrast, the biochemical and inflammatory features—including albumin (Alb), WBC, PLR, NPAR, and NLR—exhibited heterogeneous SHAP distributions with substantial overlap around zero. Their effects varied between individuals, suggesting that these laboratory parameters exerted modest but nonuniform influences on the model’s decision-making process. This variability highlights the value of SHAP visualization in capturing individual-level feature behavior that is often obscured in population-level statistical models.

Our results can also be interpreted in the context of existing machine learning studies on RA-related osteoporosis. In the study by Lee et al. (2023), models were developed using demographic factors, socioeconomic variables, comorbidities, and lifestyle information from a large national cohort. Although age and sex were similarly identified as major predictors, biochemical and disease-specific inflammatory markers—such as Alb, NLR, PLR, and NPAR—were not included in their feature set. In contrast, the present study integrates routinely measured laboratory parameters that directly reflect the inflammatory and nutritional profile of RA patients, thereby providing a disease-specific and clinically grounded risk signature. Moreover, by visualizing the heterogeneous and individualized contributions of these laboratory features through SHAP, this study offers additional interpretability not available in previous models that relied primarily on demographic and population-health variables. These distinctions underscore the novelty of our approach and highlight how combining machine learning with routine laboratory data may enhance personalized risk assessment in RA.

Rheumatoid arthritis is categorized into younger-onset (30–55 years) and elderly-onset (≥60 years) subtypes (Tan et al., 2017; Tanaka, 2021). In our cohort, the mean age of RA patients was 56.61 years, while that of RA+OP patients reached 64.60 years—consistent with the SHAP analysis identifying age as the top predictor. Bone mineral density typically peaks around age 35 and gradually declines with advancing age, with accelerated bone loss occurring after menopause in women (Long et al., 2023). The prevalence of osteoporosis rises sharply in individuals aged 65 and older (Johnston & Dagar, 2020; Pinkerton, Thomas & Dalkin, 2013), which explains why the RA+OP group, with a mean age approaching this threshold, showed a significantly higher risk. This finding underscores the need for targeted osteoporosis screening in RA patients aged 60 and above.

Regarding gender differences, SHAP analysis confirmed gender as the second-most important predictor, reflecting the dual epidemiological characteristics of RA and osteoporosis. Women are affected by RA at approximately three times the rate of men (Favalli et al., 2019), likely due to estrogen fluctuations, immunological disparities, and physiological changes related to childbirth. Postmenopausal estrogen decline not only exacerbates immune dysregulation and RA severity but also enhances osteoclast activity, accelerating bone loss—resulting in a fivefold higher osteoporosis risk in postmenopausal women compared to age-matched men (Llorente et al., 2020; Qu et al., 2023). In our cohort, female patients predominated, particularly in the RA+OP group, with a statistically significant between-group difference. This suggests an additive effect of RA-related inflammation and sex-specific physiological changes on bone loss (Ramos et al., 2025), which is strongly supported by the SHAP value’s quantification of gender’s predictive weight.

BMI, ranked third in SHAP importance and identified as a protective factor, showed distinct distribution patterns between the two groups. Both cohorts had mean BMI values within the normal range, which may be attributed to “sarcopenic obesity” in RA—where muscle mass loss is offset by fat accumulation (Novikova et al., 2019). The relationship between BMI and RA follows a U-shaped curve: obesity is associated with increased disease activity and diminished response to disease-modifying antirheumatic drugs (Curtis et al., 2019), while our SHAP analysis highlights that higher BMI (within the normal range) confers protection against osteoporosis in RA patients (Baker et al., 2015). This protective effect may be mediated by mechanical loading on bones (promoting bone formation) and the metabolic effects of adipose tissue (*e.g*., estrogen production in postmenopausal women, which mitigates bone loss). In contrast, low BMI in the RA+OP group likely reflects sustained systemic inflammation, which compromises both bone metabolism and body composition—raising the question of whether osteoporosis represents a progressive stage of RA where inflammatory burden overrides potential protective factors. This hypothesis warrants further longitudinal research to explore the dynamic relationship between BMI, inflammation, and bone health in RA.

Beyond demographic and anthropometric factors, SHAP analysis highlighted the non-negligible predictive value of inflammatory and laboratory markers, including WBC, Alb, NLR, PLR, and NPAR. As machine learning technologies advance, integrating these routinely measured parameters into clinical decision-making holds great promise for early screening and intervention (Tu et al., 2024). Previous studies have linked NLR and PLR to disease activity in various rheumatic conditions (Cheng et al., 2024; Liu et al., 2023; Mangoni & Zinellu, 2024), and associated them with osteoporosis occurrence (Chen et al., 2023; Tang et al., 2022). Suggesting potential mechanistic relevance that aligns with their predictive contribution in our model. Alb, a major plasma protein, reflects systemic health, nutritional status, and inflammatory burden (Chen et al., 2019; Zhang et al., 2025), decreased Alb levels in RA patients are closely tied to disease activity and osteoporosis risk (Liu et al., 2024; Nagayama et al., 2022), which may explain its SHAP importance (0.0299) in our model. NPAR, a novel composite index combining neutrophil percentage and albumin, has emerged as a reliable marker of systemic inflammation and disease activity (Ding et al., 2025; Zhang et al., 2025). Its inclusion in the top seven predictive features (SHAP value: 0.0243) underscores its potential as a convenient, non-invasive tool for monitoring RA-related bone health risks. While the specific direction of these markers’ impact on RA+OP prediction remains unclear in our current analysis, their consistent identification as predictive factors across clinical contexts suggests they may capture unmeasured dimensions of inflammation, nutrition, or disease severity that modulate bone health in RA—interacting with BMI’s protective effect. Further research is needed to clarify whether higher or lower values of these markers are associated with increased risk, and to explore how they intersect with BMI and other core factors in shaping osteoporosis risk (Jin et al., 2021; Jung et al., 2019).

In model development, we adopted ensemble learning to enhance predictive performance and robustness by integrating multiple base learners. All features were standardized to address differences in units and scales, and cross-validation was used to ensure consistency across datasets and reduce overfitting. Our results demonstrated that the ensemble model effectively differentiated RA from RA+OP, with strong performance across accuracy, AUC, and F1-score. Notably, SHAP value analysis not only quantified feature importance but also revealed the protective role of BMI—addressing the “black box” limitation of traditional machine learning models and enhancing clinical trust and applicability, even as the direction of influence for certain laboratory markers requires further elucidation.

## Limitations

However, this study has several limitations. First, the model was trained and validated on a relatively small dataset (*n* = 396), which increases the risk of overfitting despite the use of cross-validation and regularization strategies. The test set of approximately 80 patients still limits the stability of performance, with confidence intervals remaining wide due to the small test set size, even after bootstrapping. The limited sample size may also reduce the representativeness of the findings, as the model could capture cohort-specific patterns rather than generalizable risk signals. Larger, multicenter cohorts will be essential to validate the model, improve its stability, and enhance the reliability of feature importance estimates. In addition, although the ensemble methods demonstrated favorable performance, further refinement of model integration strategies and hyperparameter tuning is warranted to ensure robustness across diverse clinical settings and patient populations.

## Conclusions

In conclusion, we developed an ensemble learning model integrating demographic and routine laboratory parameters to differentiate patients with RA from those with RA and concomitant osteoporosis. Although the model demonstrated moderate predictive performance, these findings should be regarded as exploratory due to the limited sample size and lack of external validation. The results nonetheless highlight the potential value of routinely collected clinical data in supporting early risk stratification. Future studies incorporating larger, multicenter cohorts and independent validation datasets will be essential to determine the model’s generalizability and its potential role in clinical decision-making.

## Supplemental Information

10.7717/peerj.20852/supp-1 Supplemental Information 1 Completed STARD checklist.

10.7717/peerj.20852/supp-2 Supplemental Information 2 Code.

10.7717/peerj.20852/supp-3 Supplemental Information 3 Codebook.

10.7717/peerj.20852/supp-4 Supplemental Information 4 Anonymized data.

## Additional Information and Declarations

### Data Availability

The following information was supplied regarding data availability:

The code and measurements are available in the Supplemental Files.
