# Supplementary material for: Application of a machine learning model based on routine clinical parameters for the diagnosis of rheumatoid arthritis with concomitant osteoporosis: a retrospective study
Source: PeerJ. 2026 Feb 27;14:e20852. doi: 10.7717/peerj.20852 (PMC12951883; doi:10.7717/peerj.20852)
Supplement: Supplemental Information 3 [file peerj-14-20852-s003.docx]

Label:0= RA,1= RA&OP

Genders:0= Women,1= Men
